# Spatial patterns of leprosy in a hyperendemic state in Northern Brazil, 2001-2012

**DOI:** 10.1590/S0034-8910.2015049005866

**Published:** 2015-11-16

**Authors:** Lorena Dias Monteiro, Francisco Rogerlândio Martins-Melo, Aline Lima Brito, Carlos Henrique Alencar, Jorg Heukelbach

**Affiliations:** I Departamento de Saúde Comunitária. Faculdade de Medicina. Universidade Federal do Ceará. Fortaleza, CE, Brasil; II Secretaria de Estado da Saúde do Tocantins. Palmas, TO, Brasil; III Instituto Federal de Educação, Ciência e Tecnologia do Ceará. Caucaia, CE, Brasil; IV Division of Tropical Health and Medicine.Anton Breinl Centre for Public Health and College of Public Health, Medical and Veterinary. James Cook University. Townsville, QLD, Australia

**Keywords:** Leprosy, epidemiology, Spatial Analysis, Endemic Diseases, Neglected Diseases, Epidemiological Surveillance

## Abstract

**OBJECTIVE:**

To describe the spatial patterns of leprosy in the Brazilian state of Tocantins.

**METHODS:**

This study was based on morbidity data obtained from the *Sistema de Informações de Agravos de Notificação* (SINAN – Brazilian Notifiable Diseases Information System), of the Ministry of Health. All new leprosy cases in individuals residing in the state of Tocantins, between 2001 and 2012, were included. In addition to the description of general disease indicators, a descriptive spatial analysis, empirical Bayesian analysis and spatial dependence analysis were performed by means of global and local Moran’s indexes.

**RESULTS:**

A total of 14,542 new cases were recorded during the period under study. Based on the annual case detection rate, 77.0% of the municipalities were classified as hyperendemic (> 40 cases/100,000 inhabitants). Regarding the annual case detection rate in < 15 years-olds, 65.4% of the municipalities were hyperendemic (10.0 to 19.9 cases/100,000 inhabitants); 26.6% had a detection rate of grade 2 disability cases between 5.0 and 9.9 cases/100,000 inhabitants. There was a geographical overlap of clusters of municipalities with high detection rates in hyperendemic areas. Clusters with high disease risk (global Moran’s index: 0.51; p < 0.001), ongoing transmission (0.47; p < 0.001) and late diagnosis (0.44; p < 0.001) were identified mainly in the central-north and southwestern regions of Tocantins.

**CONCLUSIONS:**

We identified high-risk clusters for transmission and late diagnosis of leprosy in the Brazilian state of Tocantins. Surveillance and control measures should be prioritized in these high-risk municipalities.

## INTRODUCTION

Control of leprosy transmission is a difficult task in many countries, including Brazil. In 2012, approximately 233,000 new cases were reported worldwide, and this large number has mobilized governments and institutions to prioritize improvement of control measures.^12^ ^,^ ^29^ Seventeen percent of the total of leprosy cases in the world occur on the American continent; Brazil is responsible for 93.0% of these cases.^29^ In Brazil, the spatial distribution of leprosy is heterogeneous. The Northern, Midwestern and Northeastern regions Brazil have a particularly high burden of the disease.^2^ ^,^ ^22^ Most high-risk districts are concentrated in states that are part of the Brazilian Amazon, a highly endemic area.^21^ ^,^ ^22^

Tocantins state presented the second highest annual case detection rate among Brazilian states in 2012. Leprosy is still hyperendemic despite the control efforts made during recent years.^17^ ^,^ ^24^ The annual case detection rates are higher than the national mean, reaching 73.1 new cases/100,000 inhabitants in the general population and 20.8 new cases in in < 15 years-olds/100,000 inhabitants. This last indicator reflects ongoing transmission of the disease.^a^

Given the epidemiological complexity of leprosy in the Brazilian state of Tocantins and the continuously high case detection rates and transmission indicators,^2^ ^,^ ^8^ ^,^ ^15^ ^,^ ^17^ the objective of this study was to describe the spatial distribution patterns of leprosy in this hyperendemic state.

## METHODS

Tocantins is located in the northern region of Brazil and is part of the Brazilian Amazon, which has predominantly savannah-type vegetation. The territory covers an area of 277,622 km^2^, and had an estimated population of 1.4 million inhabitants in 2013. The state is administratively divided into 139 municipalities. It is located in the southeast of the Northern Brazil and borders the states of Goias (at the south), Mato Grosso (at west and southwest), Pará (at west and northwest), Maranhao (at north, northeast and east), Piaui (at east) and Bahia (at east and southeast).^b^

From 2000 to 2010, the urbanization rate in Tocantins increased from 74.3% to 79.0%, and the Municipal Human Development Index (MHDI) from 0.52 to 0.69. Extreme poverty rate in the state decreased from 22.3% to 10.2%, while income inequality, indicated by the Gini coefficient, was reduced from 0.65 to 0.60.^c^

We performed an ecological study with spatial analysis, based on new leprosy cases in Tocantins from 2001 to 2012. Spatial patterns were analyzed and high-risk areas for transmission and diagnosis of disease were identified using municipalities of residence as geographic units of analysis.

The data were obtained from the *Sistema de Informações de Agravos de Notificação* (SINAN – Notifiable Diseases Information System) from the Ministry of Health, based on compulsory notification records. These records consist of standard forms including sociodemographic and clinical information to be informed by health professionals. The database with all national notifications was obtained from the *Coordenação Geral de Hanseníase e Doenças em Eliminação* (CGHDE – General Coordination of Leprosy & Diseases in Elimination) of the Ministry of Health.

Leprosy cases are defined by the World Health Organization (WHO) as individuals who present clinical signs of the disease and require specific leprosy treatment.^d^ Records with diagnostic errors, double entries, and cases with residency in another state were excluded.

Population data were obtained from the Brazilian Institute of Geography and Statistics (IBGE). These were based on a state population census (2010) and population estimates for the other years (2001 to 2009 and 2011 to 2012).^e^

For spatial analysis, three indicators recommended by the World Health Organization (WHO) and adopted by the national program for leprosy evaluation and monitoring were analyzed: annual case detection rate in the general population (per 100,000 inhabitants), indicating the magnitude of leprosy in an area; annual case detection rate in < 15 years-olds (per 100,000 inhabitants), indicating active disease transmission; and grade 2 disability cases in the population (per 100,000 inhabitants), indicating subnotification and late diagnosis.^e^

The parameters for classification of municipalities based on the selected indicators were applied in accordance with WHO.^f^ However, due to the high values of leprosy indicators in the state, an additional parameter, called “very hyperendemic” was added for an annual case detection rate > 100 cases/100,000 inhabitants, which is far beyond the established “hyperendemic” category (> 40 cases/100 000 inhabitants). Similarly, we included for an annual case detection rate in < 15 years-olds of > 20 cases/100,000 inhabitants, and for grade 2 disability cases/100.000 inhabitants in the population of > 10 cases/100,000 inhabitants the additional parameters “very hyperendemic”. This new classification was established because almost all the municipalities showed extremely high case detection rates in all years of the study period.

The mean indicators of the period were calculated. To do so, a stable population was considered and the total number of new cases was divided by 12 years of the study. This value was divided by the population of the central year (mean of 2006 and 2007) and multiplied by 100,000. The mean indicators during the study period (2001-2012) have been used to correct random fluctuations and to reach more stable values, mainly in municipalities of small population size, i.e., with less than 20,000 inhabitants. In addition, smoothed indicators were calculated using the empirical Bayesian method. This method uses information from surrounding areas that are part of the region under study, when estimating the values for the risk areas.^4^

After descriptive spatial analysis, the presence of global spatial dependence was evaluated using the Global Moran’s I index on the smoothed indicators. Moran’s I index was applied on smoothed indicators to ensure the correction of extreme values and of areas of zero notification and/or subnotification. The method measures the correlation of a variable with itself in space. The Moran’s I index ranges from -1 to +1. Values close to zero indicate spatial randomness; positive values indicate positive spatial autocorrelation; and negative values indicate negative spatial autocorrelation.^7^

The existence of local autocorrelation (Local Index of Spatial Association – LISA) was evaluated using the local Moran’s I index.^3^ The Moran Scatter Plot was used to identify critical or transition areas, based on local Moran’s I index, to compare the value of each municipality with its neighboring municipalities and to verify spatial dependency, in addition to identifying spatial patterns.^3^ The quadrants generated in this technique are interpreted as follows: Q1 - High/High (positive values, positive means) and Q2 - Low/Low (negative values, negative means), indicating areas of positive spatial association or similar values to neighboring areas; Q3 - High/Low (positive values, negative means) and Q4 - Low/High (negative values, negative means), indicating points of negative spatial association, i.e., municipalities with values that are distinct from neighboring areas. The first two categories represent areas of agreement and the last two transition areas.^3^ Moran Maps were used for the spatial representation of the Moran Scatter Plot, considering the municipalities with statistically significant differences (p < 0.05). High-risk areas (hot spots) for disease detection, active transmission and late diagnosis were considered when categorized by municipalities with high indicator values, with other municipalities as neighbors with the same characteristics.

The software ArcGIS version 9.3 (Environmental Systems Research Institute – ESRI, Redlands, CA, USA) and TerraView version 4.1 (Instituto Nacional de Pesquisas Espaciais – INPE, Sao Jose dos Campos, SP, Brazil) were used to process, analyze and present spatial data, and to calculate the spatial autocorrelation indicators, as well as to draw thematic maps.

This study was approved by the Ethical Review Board of the Universidade Federal do Ceará (Protocol 544,962 from February 28, 2014).

## RESULTS

A total of 14,532 new cases of leprosy were notified during the study period. The mean annual case detection rate in the general population was 93.3 cases/100,000 inhabitants. The mean case detection rate in < 15 years-olds was 24.1 cases/100,000 inhabitants, and 4.2 cases/100,000 inhabitants were diagnosed with grade 2 disability.

**Figure 1:**
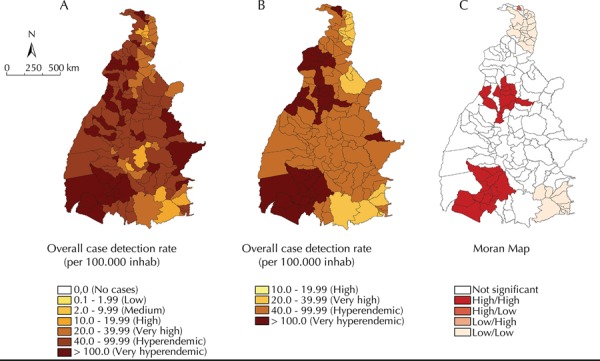
Spatial analysis of the new leprosy case detection rate (per 100,000 inhabitants): overall crude rate (A), smoothed rate by the empirical Bayesian method (B) and Moran Maps (C). Tocantins, Northern Brazil, 2001-2012.

All municipalities recorded at least one case of leprosy, and 77.0% (107/139) of the municipalities recorded hyperendemic or very hyperendemic case detection rates. The local empirical Bayesian method generated more stable smoothed indicators (Figure 1, A and B). The mean annual case detection rate reached a maximum value of 272 cases/100,000 inhabitants, while the smoothed indicator was 250.5 cases/100,000 inhabitants. The smoothed maps showed that most of the municipalities (86.3%) have a hyperendemic case detection rate that is spread over almost the entire state; there is also a cluster of municipalities in the central-north and southwest regions of the state with values ≥ 100 cases/100,000 inhabitants.

The Global Moran’s I index presented a positive and significant value (0.51; p < 0.001), which evidenced the existence of spatial dependence between the indicators in the municipalities. There were two clusters of municipalities identified with high case detection rates, which cover the central-north and southwest regions of the state. Clusters of municipalities with low detection rates were identified in the far north and southeast regions of the state (Figure 1, C).

**Figure 2:**
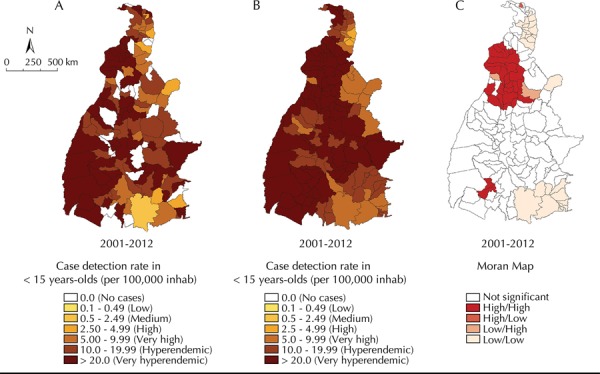
Spatial analysis of the leprosy case detection rate in < 15 years-olds (per 100,000 inhabitants): overall crude rate (A), smoothed rate by the empirical Bayesian method (B) and Moran Maps (C). Tocantins, Northern Brazil, 2001-2012.

During the period, 65.4% of the municipalities presented a hyperendemic case detection (10.0 to 19.9 cases/100,000 inhabitants) in < 15 years-olds, and 12.9% of the municipalities had no records of any cases in this age group. The Bayesian analysis identified 85.6% of the municipalities as hyperendemic. Almost the entire state territory was covered by the cluster of municipalities with very hyperendemic case detection rates in > 15 years-olds (Figure 2, A and B).

The Global Moran’s I index presented positive and significant values (0.47; p < 0.001) for the case detection rate in < 15 years-olds. Two clusters of municipalities with high detection rates were identified: the most representative involved 23 municipalities in the central-north region, and the smaller other with only two municipalities in the southwest region of the state, which is in line with the high risk areas regarding the previously evaluated indicator. Clusters of municipalities with low case detection in < 15 years-olds were identified in the extreme north and southeast regions of the state (Figure 2, C).

**Figure 3:**
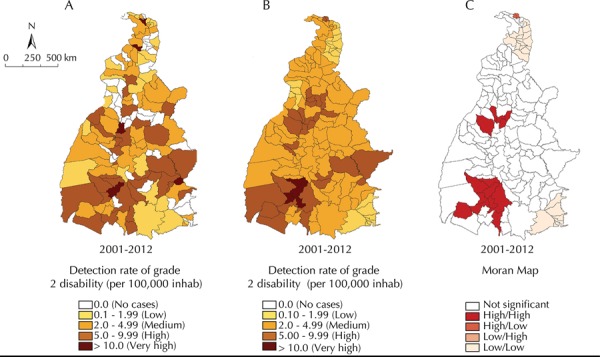
Spatial analysis of detection rate of grade 2 disability (per 100,000 inhabitants): overall crude rate (A), smoothed rate by the empirical Bayesian method (B) and Moran Maps (C). Tocantins, Northern Brazil, 2001-2012.

Among the municipalities, 26.6% presented a high detection rate of grade 2 disability cases (5.0 to 9.9 cases/100,000 inhabitants). In another 26.6% municipalities, there was no record of any cases with grade 2 physical disability (Figure 3, A). The local empirical Bayesian method identified 76.2% municipalities with a mean detection of cases (2.0 to 4.9 cases/100,000 inhabitants) (Figure 3, B). One group of municipalities in the central-north, southwest regions and some scattered municipalities in the far north and east of the state reached high levels (> 10 cases/100,000 inhabitants).

The Global Moran’s I index presented a positive and significant value (0.44; p < 0.001). Three clusters of municipalities with high detection rates were identified: the most representative cluster included nine municipalities in the southwest region; another one included five municipalities in the central-north region; and the third included two municipalities in the far north of the state. The most critical areas for this indicator coincided when referring to areas for the general case detection rate and for the detection rate in < 15 years-olds, as well as for areas with clusters of municipalities with low detection rates located in the far north and southeastern regions of Tocantins (Figure 3, C)

## DISCUSSION

The pattern of reported leprosy cases between 2001 and 2012 in the Brazilian state of Tocantins showed significant spatial heterogeneity. High-risk clusters for disease occurrence, active transmission, and late diagnosis were identified; these were mainly located in the central-north and southwest regions of the state. This study detected specific epidemiological aspects for the three analyzed indicators in Tocantins over an extended period. Our study contributed significantly to the understanding of the distribution of leprosy in the state. The approach provides data to improve leprosy control measures, to highlight operational problems and to reduce costs through targeted actions that depend on the epidemiological reality of the municipalities.

The crude indicators reflected the spatial distribution of leprosy in this state, but the thematic maps with smoothed indicators were more suitable to provide an understanding on the spatial effects caused by neighboring municipalities. The distributions indicate the spatial pattern of disease, risk areas and the influence of small populations.^4^ The spatial dependence analysis was accurate in its identification of significantly higher risk areas for the disease.

In recent studies, the identification of high-risk areas, combining different spatial analysis techniques, has enabled the analysis to become more accurate. In addition, this contributed to the definition of priority areas for specific interventions to be adopted by the control programs, as well as to the evaluation of the impact of strategic intervention measures.^2^ ^,^ ^14^ A study with different spatial approaches was performed in an area considered to be high-risk in Brazil, involving four Brazilian states, which was sufficiently valid and of paramount importance in the design of several clusters of municipalities with a high endemicity, active transmission, and diagnosis of leprosy.^1^ This spatial approach has already been applied perform effective case detection at low cost, consequently improving leprosy control measures.^26^

In our study, the geographic overlay of high-risk municipality clusters was observed in hyperendemic areas. The high values of the indicators reflect the social vulnerability of the affected populations, as well as the geographic expansion and the urbanization process, which can facilitate the maintenance and spread of the disease in the region.^9^ ^-^ ^11^ Studies regarding leprosy debate the history of the territories’ occupation as a theory to explain the persistence of foci in certain regions.^18^ ^,^ ^20^ ^,^ ^22^ In addition to these assumptions, the disease may be associated to immunity issues.^28^

The high values of the indicators of leprosy in the state of Tocantins could be caused by increasing urbanization and migration, which peaked after the construction of the national highway BR 153 (Transbrasiliana/Belém-Brasília Highway) in the 1970s and the creation of the state of Tocantins in the late 1980s.^g^ These circumstances resulted in a population growth of more than 46.0% and increasing urbanization from 25.0% to nearly 75.0% from 1970 to 2000. In 2010, practically 80.0% of its population resided in urban areas, with immigrants from all regions of Brazil.^d^ Before the BR 153 was constructed, Tocantins (a remote region north of Goias state) was considered a “white spot” on the map. The Amazon rainforest was a geographical barrier between the south and north regions of the country and hence, prevented urbanization. The region was isolated.^5^ ^,^ ^h^ The lack of exposure that the local population had to leprosy bacilli may explain the occurrence of numerous new cases after increased migration movements.^27^ It is possible that the disease had spread slowly due to access problems that prevented greater contact between people. Another possible aspect is that cases of leprosy were underreported in this isolated area.

The BR 153 restructured occupation of space in Tocantins: urban areas emerged at its margins, and migratory movements were generated.^19^ The rural exodus included mainly the poor population, including migrants from the countryside in the northeast, which is a region with low sociodemographic parameters.^5^ ^,^ ^6^ In the 1990s, all municipalities had a very low municipal human development index (MHDI) (≤ 0.499), in addition to severe social inequality (Gini coefficient: 0.63). In 2010, 42.4% of the municipalities already had a medium HDI (0.600; 0.699) and 7.9% had a high HDI (0.700; 0.799); however, the social inequality remained at a similar level (Gini coefficient: 0.60).^d^ These peculiarities suggest that the migratory and urbanization processes caused repercussions for the dynamics of health problems and were a determining factor in the epidemiological situation of leprosy in this area.

At this time, leprosy had a high endemicity in south and southeast regions in Brazil,^13^ which is where most of the migrants from Brazil’s countryside came from. Studies highlight the relationship of cases from the state of Sao Paulo with cases of the disease in the Midwestern region of Brazil.^20^ On one hand, migration could explain the transmission dynamics; on the other hand, the maintenance of endemic disease could be linked to sociodemographic and environmental factors. The risk factors that contribute to the persistence of the disease in the region can be better evaluated by using evidence found via spatial regression analysis.^9^ ^,^ ^25^

However, the better coverage by health services in the municipalities and the actions taken by the state control program in recent years may have significantly influenced the increase of these leprosy indicators. The decentralization of control actions for the municipalities, professional training, campaigns and intergovernmental partnerships can also boost detection of cases.^17^ ^,^ ^2^ ^4^ Over the short-term, a stagnation and even decline of the indicators is expected. However, the clusters of municipalities with grade 2 disability reinforces the evidence of a hidden prevalence, late diagnosis and the need to give attention to the physical rehabilitation and social aspects in these areas.^15^ ^,^ ^16^

Despite the importance of reducing the prevalence of leprosy in Brazil, some regions require intensification of surveillance actions, which is justified by the high endemicity and active transmission of the disease.^2^ ^,^ ^22^ ^,^ ^23^ ^,^ ^25^ The priorities regarding surveillance and control measures are no longer offered based on the strength of the disease’s transmission (detection rate) as well as in areas of significantly higher risk.^i^ Included in this evaluation are 24 municipalities that represented a statistically high risk for leprosy detection rate, 23 for detection in 15 years-olds and 16 for detection with grade 2 disability. However, four municipalities (Araguaina, Colinas do Tocantins, Gurupi and Palmas) were considered prioritiy areas by the national program.^24^ Different patterns are observed in the leprosy detection rate, and many regions and municipalities continue to see an increase and stabilization of the disease, which is a situation in which the application of the prevalence measurement does not fit the reality.^17^ ^,^ ^23^

The most critical areas identified by the Bayesian analysis were more extensive and covered more than half of municipalities for the detection of cases in < 15 years-olds. This situation reflects the severity of the endemic level of leprosy and early exposure to *Mycobacterium leprae.* ^1^

The presence of groups of municipalities with mean detection rates higher than expected in Tocantins evidences the persistence and strength of the disease in groups of municipalities in Midwestern and Southwestern regions of Brazil, or that health services in these regions were more efficient at detecting the cases. On the other hand, a cluster of municipalities with lower than expected mean detection coefficients in far north and southeastern regions of this state can be indicative of possible failures in health services, such as late diagnosis and cases being underreported. Municipalities with low case detection that are located near high risk areas need to strengthen their surveillance system and enhance their diagnosis and treatment procedures. Despite the advances in control activities made in recent years by the state leprosy control program in Tocantins,^24^ efforts must be made to reach lower indicators that are close to expected standards for the disease control.

This study has limitations that are related to the use of secondary data, which may show inconsistencies in relation to the quantity, quality and data processing. To minimize possible systematic errors, the national SINAN database was combined with the state SINAN database, made available by the State of Tocantins Secretariat of Health, and thereby strengthened the evidence base of this study. Despite these limitations, the results show internal consistency, coherence with existing knowledge about leprosy and are highly representative, since they included all notifications of resident cases in the state of Tocantins, even when the disease was reported in other states, from 2001 to 2012.

The results of this study demonstrate that leprosy is a persistent public health problem in Tocantins, with higher risk in the identified clusters. There is active disease transmission, with high indicators, wide geographical distribution and there are significant regional differences, despite the actions taken by the control program. The epidemiological framework’s continuity can be influenced by the occupational process and by the migratory origin of the territory, or even by the existence of areas with different vulnerabilities to the social production of the disease. Clusters of high-risk municipalities were identified in a territory that was recognized as hyperendemic, which had adopted indicators that evaluate health services and the dynamics of the disease’s transmission. These findings highlight the need for new research approaches so that the conditions and determinants of the disease can be better understood. It is possible that the regional disparities of the detection rates will remain even when the official elimination target has been reached.

## Footnotes

a Dados disponibilizados após solicitação via portal da transparência. Ministério da Saúde, Secretaria de Vigilância em Saúde, Coordenação Geral de Hanseníase e Doenças em Eliminação. Brasília (DF); 2013 [cited 2013 Sept 5]. Banco de dados concedido por solicitação no portal da transparência. Available from: http://portalsaude.saude.gov.br/index.php/o-ministerio/principal/saude-com-mais-transparencia

b Instituto Brasileiro de Geografia e Estatística. Unidades da Federação: Tocantins. Rio de Janeiro (RJ); 2014 [cited 2014 Mar 12]. Available from: http://www.ibge.gov.br/estadosat/perfil.php?sigla=to Lista?

c Programa das Nações Unidas para o Desenvolvimento; Instituto de Pesquisa Econômica Aplicada; Fundação João Pinheiro. Atlas do desenvolvimento humano no Brasil 2013. Brasília (DF); 2013 [cited 2014 Mar 12]. Available from: http://atlasbrasil.org.br/2013/

d Ministério da Saúde. Portaria nº 3.125, de 7 de outubro de 2010. Aprova as diretrizes para vigilância, atenção e controle da hanseníase. *Diario Oficial Uniao.* 27 mar 2010; Seção 1:55-60.

e Ministério da Saúde. Coordenação Geral de Hanseníase e Doenças em Eliminação. Banco de dados concedido por solicitação no portal da transparência. Brasília (DF); 2013 [cited 2014 Mar 12]. Available from: http://tabnet.datasus.gov.br/cgi/deftohtm.exe?ibge/cnv/popto.def

f World Health Organization, Regional Office for South-East Asia. Enhanced global strategy for further reducing the disease burden due to leprosy (plan period: 2011-2015). New Delhi; 2009 [cited 2015 May 9]. Available from: http://www.searo.who.int/entity/global_leprosy_programme/documents/enhanced_global_strategy_2011_2015.pdf

g Ribeiro FA. A invenção do Tocantins: memória, história e representação [dissertation]. Goiânia (GO): Faculdade de Ciências Humanas e Filosofia da Universidade Federal de Goiás; 2001.

h Andrade RP. Conceitos de progresso e natureza na construção da Belém-Brasília. In: Anais do 13 Seminário Nacional de História da Ciência e Tecnologia; 2012; São Paulo, Brasil. Rio de Janeiro: Sociedade Brasileira da História da Ciência; 2012 [cited 2015 May 9]. p.1-9. Available from: http://www.sbhc.org.br/resources/anais/10/1344996001_ARQUIVO_SBHC2012.pdf

i Ministério da Saúde, Secretaria de Vigilância em Saúde, Departamento de Vigilância das Doenças Transmissíveis. Plano integrado de ações estratégicas de eliminação da hanseníase, filariose, esquistossomose e oncocercose como problema de saúde pública, tracoma como causa de cegueira e controle das geohelmintíases: Plano de Ação 2011-2015. Brasília (DF); 2012 [cited 2015 May 9]. (Série C. Projetos, Programas e Relatórios). Available from: http://bvsms.saude.gov.br/bvs/publicacoes/plano_integrado_acoes_estrategicas_2011_2015.pdf

## References

[1] Alencar CH, Barbosa JC, Ramos NA, Alencar MJF, Pontes RJS, Castro CGJ (2008). Hanseníase no município de Fortaleza, CE, Brasil: aspectos epidemiológicos e operacionais em menores de 15 anos (1995-2006). Rev Bras Enferm.

[2] Alencar CH, Ramos AN, Santos ES, Richter J, Heukelbach J (2012). Clusters of leprosy transmission and of late diagnosis in a highly endemic area in Brazil: focus on different spatial analysis approaches. Trop Med Int Health.

[3] Anselin L (1995). Local indicators of spatial association−LISA. Geogr Anal.

[4] Assunção RM, Barreto SM, Guerra HL, Sakurai E (1998). Mapas de taxas epidemiológicas: uma abordagem Bayesiana. Cad Saude Publica.

[5] Becker BK (1977). A implantação da rodovia Belém-Brasília e o desenvolvimento regional. Anu Inst Geocienc.

[6] Borges BG (2002). A Rodovia Belém-Brasília. Rev Educ Mudança.

[7] Cliff AD, Ord JK (1981). Spatial processes: models & applications.

[8] Heukelbach J, Chichava OA, Oliveira AR, Häfner K, Walther F, Alencar CHM (2011). Interruption and defaulting of multidrug therapy against leprosy: population-based study in Brazil’s Savannah Region. PLoS Negl Trop Dis.

[9] Imbiriba ENB, Silva AL, Souza WV, Pedrosa V, Cunha MG, Garnelo L (2009). Social inequality, urban growth and leprosy in Manaus: a spatial approach. Rev Saude Publica.

[10] Kerr-Pontes LRS, Barreto ML, Evangelista CM, Rodrigues LC, Heukelbach J, Feldmeier H (2006). Socioeconomic, environmental, and behavioural risk factors for leprosy in North-east Brazil: results of a case-control study. Int J Epidemiol.

[11] Lapa T, Ximenes R, Silva NN, Souza W, Albuquerque MFM, Campozana G (2001). Vigilância da hanseníase em Olinda, Brasil, utilizando técnicas de análise espacial. Cad Saude Publica.

[12] Lockwood DNJ, Suneetha S (2005). Leprosy: too complex a disease for a simple elimination paradigm. Bull World Health Organ.

[13] Lombardi C (1984). Aspectos epidemiológicos da mortalidade entre doentes de hanseniase no Estado de São Paulo, Brasil (1931-1980). Rev Saude Publica.

[14] Martins-Melo FR, Lima MS, Ramos AN, Alencar CH, Heukelbach J (2014). Mortality and case fatality due to visceral leishmaniasis in Brazil: a nationwide analysis of epidemiology, trends and spatial patterns. PloS One.

[15] Monteiro LD, Alencar CHM, Barbosa JC, Braga KP, Castro MD, Heukelbach J (2013). Incapacidades físicas em pessoas acometidas pela hanseníase no período pós-alta da poliquimioterapia em um município no Norte do Brasil. Cad Saude Publica.

[16] Monteiro LD, Alencar CH, Barbosa JC, Novaes CCBS, Siilva RCP, Heukelbach J (2014). Limited activity and social participation after hospital discharge from leprosy treatment in a hyperendemic area in north Brazil. Rev Bras Epidemiol.

[17] Monteiro LD, Martins-Melo FR, Brito AL, Silveira ML, Alencar CH, Heukelbach J (2015). Tendências da hanseníase no Tocantins, um estado hiperendêmico do Norte do Brasil, 2001-2012. Cad Saude Publica.

[18] Montenegro ACD, Werneck GL, Kerr-Pontes LRS, Barreto ML, Feldmeier H (2004). Spatial analysis of the distribution of leprosy in the State of Ceará, Northeast Brazil. Mem Inst Oswaldo Cruz.

[19] Murto C, Ariza L, Alencar CH, Chichava AO, Oliveira AR, Kaplan C (2014). Migration among individuals with leprosy: a population-based study in Central Brazil. Cad Saude Publica.

[20] Opromolla PA, Dalben I, Cardim M (2006). Análise geoestatística de casos de hanseníase no Estado de São Paulo, 1991-2002. Rev Saude Publica.

[21] Penna MLF, Oliveira MLWR, Penna G (2009). Spatial distribution of leprosy in the Amazon region of Brazil. Emerg Infect Dis.

[22] Penna MLF, Oliveira MLWR, Penna GO (2009). The epidemiological behaviour of leprosy in Brazil. Lepr Rev.

[23] Rodrigues LC, Lockwood DNJ (2011). Leprosy now: epidemiology, progress, challenges, and research gaps. Lancet Infect Dis.

[24] Secretaria de Estado da Saúde do Tocantins, Coordenação de Doenças Transmissíveis e Não Transmissíveis (2013). Bol Epidemiol Hansen.

[25] Silva DRX, Ignotti E, Souza-Santos R, Hacon SS (2010). Hanseníase, condições sociais e desmatamento na Amazônia brasileira. Rev Panam Salud Publica.

[26] De Souza Dias MC, Dias GH, Nobre ML (2007). The use of Geographical Information System (GIS) to improve active leprosy case finding campaigns in the municipality of Mossoró, Rio Grande do Norte State, Brazil. Lepr Rev.

[27] Talhari S, Aguiar AP, Matos TT, Spener S, Borborema CAT (1981). Hanseniase no Estado do Amazonas: histórico e desativação do leprosário. An Bras Dermatol.

[28] van Beers SM, de Wit MYL, Klatser PR (1996). The epidemiology of Mycobacterium leprae: recent insight. FEMS Microbiol Lett.

[29] World Health Oganization (2014). Global leprosy situation. Wkly Epidemiol Rec.

