# Supplementary material for: Spatial patterns of leprosy in a hyperendemic state in Northern Brazil, 2001-2012
Source: Rev Saude Publica. 2015 Nov 16;49:84. doi: 10.1590/S0034-8910.2015049005866 (PMC4650934; doi:10.1590/S0034-8910.2015049005866)
Supplement: Translation

# Translation: Padrões espaciais da hanseníase em um estado hiperendêmico no Norte do Brasil, 2001-2012

**Authors:** Lorena Dias Monteiro, Francisco Rogerlândio Martins-Melo, Aline Lima Brito, Carlos Henrique Alencar, Jorg Heukelbach

**Affiliations:** I Departamento de Saúde Comunitária. Faculdade de Medicina. Universidade Federal do Ceará. Fortaleza, CE, Brasil; II Secretaria de Estado da Saúde do Tocantins. Palmas, TO, Brasil; III Instituto Federal de Educação, Ciência e Tecnologia do Ceará. Caucaia, CE, Brasil; IV Division of Tropical Health and Medicine.Anton Breinl Centre for Public Health and College of Public Health, Medical and Veterinary. James Cook University. Townsville, QLD, Australia

**Keywords:** Hanseníase, epidemiologia, Análise Espacial, Doenças Endêmicas, Doenças Negligenciadas, Vigilância Epidemiológica

## Abstract

**OBJETIVO:**

**:** Descrever os padrões espaciais da hanseníase no estado do Tocantins.

**MÉTODOS:**

**:** Estudo baseado em dados de morbidade obtidos do Sistema de Informações de Agravos de Notificação (Sinan), do Ministério da Saúde. Foram incluídos todos os casos novos de hanseníase residentes no estado do Tocantins, entre 2001 a 2012. Além da descrição dos indicadores gerais da doença, foi realizada análise espacial descritiva, análise bayesiana empírica local e análise de dependência espacial por meio dos índices de Moran global e local.

**RESULTADOS:**

**:** Foram registrados 14.532 casos novos no período estudado. Pelo coeficiente de detecção geral, 77,0% dos municípios foram classificados como hiperendêmicos (> 40 casos/100.000 habitantes). Para a detecção em menores de 15 anos, 65,4% foram hiperendêmicos (10,0 a 19,9 casos/100 mil habitantes), e 26,6% apresentaram detecção com grau 2 de incapacidade entre 5,0 e 9,9 casos/100 mil habitantes. Houve sobreposição geográfica de aglomerados de municípios com elevados coeficientes de detecção em áreas hiperendêmicas. Foram identificados aglomerados de alto risco para ocorrência (índice global de Moran: 0,51; p < 0,001), transmissão ativa (0,47; p < 0,001) e diagnóstico tardio da doença (0,44; p < 0,001), localizados principalmente nas regiões centro-norte e sudoeste do estado.

**CONCLUSÕES:**

**:** Existem aglomerados de elevado risco para transmissão e diagnóstico tardio da hanseníase no estado do Tocantins. As medidas de vigilância e controle devem ser priorizadas nos municípios de alto risco identificados.

## INTRODUÇÃO

O controle na transmissão da hanseníase é difícil de ser alcançado em muitos países, incluindo no Brasil. Aproximadamente 233 mil casos novos foram registrados no mundo em 2012 e esse elevado número mobiliza governos e instituições para o seu controle.^12^ ^,^ ^29^ Do total de casos de hanseníase no mundo, o continente americano agrega quase 17,0%, dos quais o Brasil é responsável por 93,0%.^29^ A distribuição espacial da hanseníase é heterogênea no Brasil. As regiões Norte, Centro-Oeste e Nordeste apresentam alta carga da doença.^2^ ^,^ ^22^ A maioria dos municípios com alto risco concentra-se em estados que compõem a Amazônia brasileira, território altamente endêmico.^21^ ^,^ ^22^

Tocantins ocupou o segundo lugar entre os estados brasileiros em casos novos por 100 mil habitantes em 2012. A hanseníase permanece hiperendêmica apesar dos esforços dos programas de controle nos últimos anos.^17^ ^,^ ^24^ Os coeficientes de detecção anuais são maiores que a média nacional, com valores que chegaram a 73,1/100 mil habitantes na população geral e a 20,8/100 mil habitantes em menores de 15 anos. Este indicador está relacionado à força de transmissão da doença.^a^

Dada a complexidade epidemiológica da hanseníase no estado do Tocantins e a manutenção de elevados coeficientes de detecção e transmissão da doença,^2^ ^,^ ^8^ ^,^ ^15^ ^,^ ^17^ o objetivo deste estudo foi descrever os padrões espaciais da distribuição da hanseníase em estado hiperendêmico.

## MÉTODOS

Tocantins é localizado na região Norte do Brasil e faz parte da Amazônia brasileira com vegetação predominantemente de cerrado. Possui extensão territorial de 277.622 km^2^, com população estimada de 1.4 milhões de habitantes, em 2013. Composto por 139 municípios, o estado é localizado a sudeste da região Norte e tem como limites os estados de Goiás ao Sul, Mato Grosso a oeste e sudoeste, Pará a oeste e noroeste, Maranhão ao norte, nordeste e leste, Piauí ao leste e Bahia ao leste e sudeste.^b^

Entre 2000 e 2010, a taxa de urbanização do Tocantins subiu de 74,3% para 79,0%, e o Índice de Desenvolvimento Humano Municipal (IDH), de 0,52 para 0,69. A extrema pobreza decresceu de 22,3% para 10,2% e a desigualdade de renda, apontada pelo índice de Gini, reduziu de 0,65 para 0,60.^c^

Foi realizado estudo ecológico com análise espacial utilizando os casos novos de hanseníase residentes no Tocantins de 2001 a 2012. Foram analisados os padrões espaciais e identificadas as áreas de alto risco para transmissão e diagnóstico tardio da doença utilizando os municípios de residência como unidade geográfica de análise.

Os dados foram obtidos do Sistema Nacional de Agravos de Notificação (Sinan) do Ministério da Saúde, provenientes das fichas de notificações compulsórias. Essas fichas são formulários padronizados com informações sociodemográficas e clínicas preenchidas por profissionais de saúde. O banco de dados com todas as notificações nacionais foi obtido junto à Coordenação Geral de Hanseníase e Doenças em Eliminação do Ministério da Saúde (CGHDE-MS).

Caso de hanseníase foi definido pela Organização Mundial de Saúde (OMS) como a pessoa que apresenta sinais clínicos da doença e requeira tratamento específico de hanseníase.^d^ Foram excluídos os registros com erro de diagnóstico, duplicidades, município ignorado e casos residentes em outros estados.

Dados populacionais foram obtidos do Instituto Brasileiro de Geografia e Estatística (IBGE) com base em dados dos censos da população do estado (2010) e estimativas populacionais para os anos intercensitários (2001 a 2009 e 2011 a 2012).^e^

Para a análise espacial, foram selecionados três indicadores preconizados pela Organização Mundial da Saúde (OMS) e adotados pelo programa nacional para avaliação e monitoramento da hanseníase: coeficiente de detecção de casos novos na população geral (por 100 mil habitantes), que indica a magnitude da hanseníase em uma área; coeficiente de detecção em menores de 15 anos (por 100 mil habitantes), que indica a transmissão ativa da doença; e número de casos novos com grau 2 de incapacidade detectados em uma população (por 100 mil habitantes), indicando subdetecção e diagnóstico tardio.^e^

Os parâmetros para os indicadores selecionados foram aplicados conforme estabelecido pela OMS.^f^ No entanto, devido aos elevados valores dos indicadores da hanseníase no estado, foi adicionada nova classificação, denominada “muito hiperendêmico”, englobando o valor de detecção geral superior a 100 casos/100 mil habitantes, acima da categoria “hiperendêmica” (valores acima de 40 casos/100 mil habitantes). O mesmo critério foi estabelecido para o indicador de detecção em menores de 15 anos. A nova classificação “muito hiperendêmico” foi aplicada para valores superiores a 20 casos/100 mil habitantes, enquanto a nova classificação foi obtida para valores superiores a 10 casos/100 mil para o grau 2 de incapacidade física. Essa nova classificação foi estabelecida porque quase todos os municípios apresentaram coeficientes de detecção extremamente elevados em todos os anos do período.

Para cada um desses indicadores, foi realizado o cálculo do indicador médio para o período. Foi estabelecida a população estável e somados os números de casos novos, divididos pelos 12 anos do estudo. Esse valor foi dividido pela população do ano central (média dos anos de 2006 e 2007) e multiplicada por 100 mil.

Os indicadores foram calculados obtendo-se a média do período total (2001 a 2012) para corrigir flutuações aleatórias e proporcionar maior estabilidade nos indicadores, principalmente em municípios com pequenas populações, i.e., com menos de 20 mil habitantes. Além disso, foram calculados indicadores suavizados pelo método bayesiano empírico local, que, ao estimar o risco de área, usa informações de áreas vizinhas que formam a região em estudo.^4^

Após a análise espacial descritiva, foi avaliada a presença de dependência espacial global, utilizando-se o índice Global de Moran I sobre os indicadores suavizados. O índice de Moran I foi aplicado nos indicadores suavizados para garantir a correção de valores extremos e de áreas silenciosas da doença. O método mede a correlação de uma variável com ela mesma no espaço. O índice de Moran I varia de -1 a +1. Valores próximos de zero indicam aleatoriedade espacial; valores positivos indicam autocorrelação espacial positiva; e valores negativos indicam autocorrelação espacial negativa.^7^

Avaliou-se a existência de autocorrelação local (*Local Index of Spatial Association* – LISA) por meio do índice de Moran local.^3^ O Diagrama de Espalhamento de Moran foi utilizado para identificar as áreas críticas ou de transição, com base no Índice de Moran local, a fim de comparar o valor de cada município com os municípios vizinhos e verificar dependência espacial, além de identificar os padrões espaciais.^3^ Os quadrantes gerados nessa técnica são interpretados da seguinte forma: Q1 - Alto/Alto (valores positivos, médias positivas) e Q2 - Baixo/Baixo (valores negativos, médias negativas), indicando pontos de associação espacial positiva ou similares aos vizinhos; Q3 - Alto/Baixo (valores positivos, médias negativas) e Q4 - Baixo/Alto (valores negativos, médias negativas), indicando pontos de associação espacial negativa, i.e., municípios que possuem valores distintos dos seus vizinhos. As duas primeiras categorias representam áreas de concordância e as duas últimas áreas de transição.^3^ *Moran Maps* foram usados para a representação espacial do Diagrama de Espalhamento de Moran, considerando os municípios com diferenças estatisticamente significantes (p < 0,05). Áreas de alto risco (*hot-spots*) para a detecção da doença, transmissão ativa e diagnóstico tardio foram consideradas quando formadas pelos municípios com elevados valores dos indicadores, tendo como vizinhos outros municípios com a mesma característica.

Os *softwares* ArcGIS versão 9.3 (*Environmental Systems Research Institute* – ESRI, Redlands, CA, USA) e TerraView versão 4.1 (Instituto Nacional de Pesquisas Espaciais – INPE, São José dos Campos, SP, Brasil) foram utilizados para o processamento, análise, apresentação de dados cartográficos e cálculo dos indicadores de autocorrelação espacial, bem como para a construção de mapas temáticos.

Este estudo foi aprovado pelo Comitê de Ética em Pesquisa (CEP) da Universidade Federal do Ceará (Parecer 544.962 de 28/2/2014).

## RESULTADOS

Foram registrados 14.532 casos novos de hanseníase no período. O coeficiente médio anual de detecção de casos novos na população geral foi de 93,3 casos/100 mil habitantes. Na população menor de 15 anos, foi de 24,1 casos/100 mil habitantes e foram diagnosticados 4,2 casos com grau 2 de incapacidade física/100 mil habitantes.

Todos os municípios registraram casos de hanseníase e 77,0% (107/139) registraram coeficiente de detecção geral hiperendêmico. O método bayesiano empírico local gerou indicadores corrigidos mais estáveis (Figura 1, A e B). O coeficiente de detecção bruto médio anual atingiu valor máximo de 272 casos/100 mil habitantes, enquanto o indicador suavizado foi de 250,5 casos/100 mil habitantes. Os mapas suavizados mostraram a maioria dos municípios (86,3%) com coeficiente de detecção hiperendêmico distribuídos por quase todo o estado, e um aglomerado de municípios na região centro-norte e sudoeste do estado com valores acima de 100 casos/100 mil habitantes.

**Figura 1:** Análise espacial do coeficiente de detecção de casos novos de hanseníase (por 100.000 habitantes): coeficiente bruto (A), coeficiente suavizado pelo método bayesiano empírico local (B) e *Moran Maps* (C). Tocantins, 2001-2012.

O índice Global de Moran I apresentou valor positivo e significativo (0,51; p < 0,001), apontando a existência de dependência espacial entre os indicadores nos municípios. Foram identificados dois aglomerados (*clusters*) de municípios com elevados coeficientes de detecção, abrangendo as regiões centro-norte e sudoeste do estado. *Clusters* de municípios com baixos coeficientes de detecção foram identificados no extremo norte e sudeste do estado (Figura 1, C).

Durante o período, 65,4% dos municípios apresentaram coeficiente hiperendêmico (10,0 a 19,9 casos/100 mil habitantes) para menores de 15 anos e 12,9% dos municípios não apresentaram registro de casos. A análise bayesiana identificou 85,6% de municípios hiperendêmicos. O aglomerado de municípios na classificação muito hiperendêmica para a detecção em crianças cobriu quase todo o território (Figura 2, A e B).

**Figura 2:** Análise espacial do coeficiente de detecção de casos novos de hanseníase em menores de 15 anos de idade (por 100.000 habitantes): coeficiente bruto (A), coeficiente suavizado pelo método bayesiano empírico local (B) e *Moran Maps* (C). Tocantins, 2001-2012.

O índice Global de Moran I apresentou valores positivos e significativos (0,47; p < 0,001) para o coeficiente de detecção em criança. Foram identificados dois *clusters* de municípios com coeficientes de detecção elevados: o mais representativo envolveu 23 municípios na região centro-norte, e outro menor com apenas dois municípios na região sudoeste do estado, o que corrobora com as áreas mais críticas quanto ao indicador avaliado anteriormente. *Clusters* de municípios com baixos coeficientes de detecção foram identificados no extremo norte e sudeste do estado (Figura 2, C).

Dos municípios, 26,6% apresentaram alta detecção de casos com incapacidades físicas visíveis (5,0 a 9,9 casos/100 mil habitantes). Em 26,6%, não houve registro de casos com grau 2 de incapacidade física (Figura 3, A). O método bayesiano empírico local identificou 76,2% dos municípios com detecção média (2,0 a 4,9 casos/100 mil habitantes) (Figura 3, B). Um grupo de municípios na região centro-norte, sudoeste e alguns dispersos no extremo norte e leste do estado atingiram patamares altos (> 10 casos/100 mil habitantes).

**Figura 3:** Análise espacial do coeficiente de detecção de casos novos de hanseníase com grau 2 de incapacidade física (por 100.000 habitantes): coeficiente bruto (A), coeficiente suavizado pelo método bayesiano empírico local (B) e *Moran Maps* (C). Tocantins, 2001-2012.

O índice Global de Moran I apresentou valor positivo e significativo (0,44; p < 0,001). Foram identificados três *clusters* de municípios com altos coeficientes de detecção: um mais representativo, envolvendo nove municípios na região sudoeste; outro menor, envolvendo cinco municípios na região centro-norte; e um terceiro, com dois municípios no extremo norte. As áreas mais críticas para esse indicador coincidiram quanto às áreas para o coeficiente de detecção geral e de detecção em menores de 15 anos, bem como para as áreas com *clusters* de municípios com baixos coeficientes de detecção localizados no extremo norte e sudeste do estado (Figura 3, C).

## DISCUSSÃO

O padrão de casos de hanseníase notificados entre 2001 e 2012 no estado do Tocantins mostrou heterogeneidade espacial significativa. Foram identificados aglomerados de alto risco para ocorrência, transmissão ativa e diagnóstico tardio da doença, localizados principalmente nas regiões centro-norte e sudoeste do estado. As análises aplicadas identificaram as áreas críticas para os três indicadores analisados. Entretanto, este estudo detectou mais detalhadamente os aspectos específicos para Tocantins durante um período prolongado.

A abordagem aplicada é importante para a compreensão da distribuição da doença no estado. Oferece subsídios consistentes para o aperfeiçoamento das atividades de controle da transmissão da hanseníase, para o apontamento de problemas operacionais e ainda para a redução de custos por meio de ações direcionadas conforme a realidade epidemiológica dos municípios.

Embora as distribuições dos indicadores brutos traduzam a expressão espacial da hanseníase no estado, os mapas temáticos com os indicadores suavizados foram mais apropriados para a compreensão dos efeitos espaciais ocasionados pelos municípios vizinhos mais próximos. Permitiram visualizar melhor o padrão espacial da doença, os espaços de risco e a influência de pequenas populações.^4^ A análise de dependência espacial foi precisa por confirmar a identificação de áreas significativamente de maior risco da doença.

Em estudos recentes, a identificação de áreas de alto risco, por meio de combinação de diferentes técnicas de análise espacial, possibilitou melhor precisão na análise. Além disso, contribuiu para o delineamento de áreas prioritárias para intervenções específicas a serem adotadas pelos programas de controle, bem como avaliação dos impactos das ações estratégicas.^2^ ^,^ ^14^ Estudo com diferentes abordagens espaciais foi realizado em área considerada de alto risco no Brasil, envolvendo quatro estados brasileiros, e foi suficientemente válido e de suma importância no delineamento de vários *clusters* de municípios com elevada magnitude, transmissão ativa e diagnóstico tardio de hanseníase.^1^ A abordagem espacial já foi aplicada para direcionar a busca ativa de casos com estratégias eficazes e de baixo custo para controlar a hanseníase.^26^

Neste estudo, a sobreposição geográfica de aglomerados de municípios de alto risco foi observada em áreas hiperendêmicas. Os altos valores dos indicadores refletem a vulnerabilidade social da doença, bem como a expansão geográfica e o processo de urbanização, que podem favorecer a circulação e manutenção da doença na região.^9^ ^-^ ^11^ Estudos sobre a hanseníase debatem a história da ocupação dos territórios como teoria da persistência dos focos de infecção em algumas regiões.^18^ ^,^ ^20^ ^,^ ^22^ Além desses pressupostos, a doença pode estar associada às questões de imunidade.^28^

Os elevados valores dos indicadores da hanseníase no estado do Tocantins podem ser resultantes de um processo crescente de urbanização e migração, potencializado após a construção da BR 153 (rodovia transbrasiliana/Belém-Brasília) na década 1970 e a sua criação no final da década de 1980.^g^ Essas circunstâncias resultaram em crescimento populacional de mais de 46,0% e na elevação do grau de urbanização de 25,0% para quase 75,0% entre 1970 e 2000. Praticamente, em 2010, 80,0% da população residia na zona urbana, com imigrantes de todas as regiões do País.^d^ Antes da construção da rodovia, Tocantins (antiga região norte de Goiás) era considerado um “vazio” demográfico. A floresta amazônica impedia a ligação entre o Sul e o Norte do País e, consequentemente, a urbanização. Havia um isolamento nessa região.^5^ ^,^ ^h^ A falta de exposição da população local à infecção explicaria a ocorrência de numerosos casos novos.^27^ Possivelmente, a doença se disseminou lentamente devido aos problemas de acesso que antes impediam maior contato entre as pessoas. Outro aspecto é a possível subnotificação da hanseníase nessa área isolada.

A rodovia reestruturou a ocupação do espaço em Tocantins: aglomerados urbanos surgiram às suas margens, e gerou movimentos migratórios.^19^ O êxodo rural foi marcante por parte da população pobre, inclusive por migrantes do interior do Nordeste, compondo uma região marcada por desfavoráveis questões sociais.^5^ ^,^ ^6^ Na década de 1990, todos os municípios apresentavam índice de desenvolvimento humano municipal (IDHM) muito baixo (≤ 0,499), além de acentuada desigualdade social (Índice de Gini: 0,63). Em 2010, 42,4% dos municípios já estavam com IDH médio (0,600;0,699) e 7,9%, alto (0,700;0,799); entretanto, a desigualdade permaneceu estagnada (Índice de Gini: 0,60).^d^ Essas particularidades sugerem que os processos migratórios e de urbanização repercutiram na dinâmica dos problemas de saúde e foram determinantes na situação epidemiológica da doença nesse espaço.

Nessa época, a hanseníase apresentava alta endemicidade nas regiões Sul e Sudeste do Brasil,^13^ de onde partia a maioria dos migrantes para exploração do interior do Brasil. Estudos apontam a relação de casos oriundos do estado de São Paulo com a ocorrência de casos da doença na região Centro-Oeste do País.^20^ Por um lado, a migração poderia explicar a dinâmica de transmissão; por outro, a manutenção da endemia poderia estar associada a fatores socioeconômicos e ambientais. Os fatores de risco que contribuem para a persistência da infecção na região podem ser mais bem avaliados por meio de evidências encontradas em uma análise de regressão espacial.^9^ ^,^ ^25^

Contudo, a melhor cobertura dos serviços de saúde nos municípios e as ações desenvolvidas pelo programa de controle estadual nos últimos anos podem ter influenciado significativamente o incremento desses elevados indicadores. A descentralização das ações de controle para os municípios, treinamento de profissionais, campanhas e parcerias intergovernamentais também podem impulsionar a detecção de casos.^17^ ^,^ ^24^ Espera-se a estagnação e até diminuição dos indicadores em curto prazo. Entretanto, os aglomerados de municípios com grau 2 reforçam a evidência de prevalência oculta nessas áreas, diagnóstico tardio e a necessidade de atenção nos aspectos de reabilitação física e social.^15^ ^,^ ^16^

Apesar da importante redução da prevalência da hanseníase no Brasil, algumas regiões demandam intensificação das ações de vigilância, justificadas por um padrão de alta endemicidade e transmissão ativa da doença.^2^ ^,^ ^22^ ^,^ ^23^ ^,^ ^25^ As prioridades às medidas de vigilância e controle deixaram de ser ofertadas com base na força de transmissão da doença (coeficiente de detecção), bem como nas áreas significativamente de maior risco.^i^ Nesta avaliação, 24 municípios representaram risco estatisticamente alto para hanseníase na detecção geral, 23 para detecção em menor de 15 anos e 16 para a detecção com grau 2 de incapacidade. No entanto, quatro municípios (Araguaína, Colinas do Tocantins, Gurupi e Palmas) foram considerados prioritários pelo programa nacional.^24^ Observam-se padrões diferenciados no coeficiente de detecção da hanseníase, e muitas regiões e municípios continuam com aumento e estabilidade da doença, situação em que a aplicação da medida de prevalência não condiz com a realidade epidemiológica.^17^ ^,^ ^23^

As áreas mais críticas identificadas pela análise bayesiana foram mais extensas e cobriram mais da metade dos municípios para o indicador de detecção de casos em crianças. O cenário reflete a gravidade do nível endêmico da hanseníase e exposição precoce a *Mycobacterium leprae.* ^1^

A presença de grupos de municípios com coeficientes de detecção médios acima do esperado no Tocantins sinaliza a persistência e maior força da doença nos grupos de municípios das regiões centro-norte e sudoeste, ou ainda, que os serviços de saúde dessas regiões foram mais eficientes na detecção de casos. Por outro lado, aglomerado de municípios com coeficientes médios de detecção abaixo do esperado nas regiões do extremo norte e sudeste do estado pode ser indicativo de possíveis falhas nos serviços de saúde, como o diagnóstico tardio e a subnotificação de casos. Municípios com baixa detecção de casos e localizados próximos às áreas de alto risco precisam fortalecer o sistema de vigilância e intensificar o diagnóstico e tratamento. Apesar dos avanços nas ações de controle realizadas nos últimos anos pelo programa estadual de controle da hanseníase no Tocantins,^24^ deve-se empreender esforços para alcançar indicadores mais baixos e próximos aos padrões esperados para o seu controle.

Este estudo apresenta limitações decorrentes da utilização de dados secundários, que podem apresentar inconsistência em relação à quantidade, qualidade e processamento das informações. Para minimizar possíveis erros sistemáticos, o banco de dados do Sinan nacional foi confrontado com os dados do banco do Sinan estadual, disponibilizado pela Secretaria de Estado da Saúde do Tocantins, fortalecendo a base de evidências desse estudo. Apesar dessas limitações, os resultados mostram consistência interna, coerência com conhecimentos existentes sobre a hanseníase e são altamente representativos, já que incluíram todas as notificações de casos residentes no estado do Tocantins, mesmo quando notificados em outras unidades federativas de 2001 a 2012.

Os resultados desse estudo evidenciam a persistência da hanseníase como problema de saúde pública no Tocantins, com maior risco em aglomerados delimitados identificados. A transmissão da doença é ativa, com elevados indicadores, ampla distribuição geográfica e diferenças regionais importantes, apesar das ações desenvolvidas pelo programa de controle. A sustentação do quadro epidemiológico pode ser influenciada pelo processo de ocupação e origem migratória do território, ou pela existência de áreas de diferentes vulnerabilidades à produção social da doença. Foram identificados *clusters* de municípios de alto risco em um território reconhecidamente hiperendêmico, com adoção de indicadores que avaliaram os serviços de saúde e a dinâmica de transmissão da doença. Esses achados apontam a necessidade de novas abordagens de pesquisa para melhor compreensão dos fatores condicionantes e determinantes da doença. Possivelmente, as disparidades regionais dos coeficientes de detecção permanecerão mesmo com a meta de eliminação alcançada.

## Footnotes

a Dados disponibilizados após solicitação via portal da transparência. Ministério da Saúde, Secretaria de Vigilância em Saúde, Coordenação Geral de Hanseníase e Doenças em Eliminação. Brasília (DF); 2013 [citado 2013 set 5]. Banco de dados concedido por solicitação no portal da transparência. Disponível em: http://portalsaude.saude.gov.br/index.php/o-ministerio/principal/saude-com-mais-transparencia

b Instituto Brasileiro de Geografia e Estatística. Unidades da Federação: Tocantins. Rio de Janeiro (RJ); 2014 [citado 2014 mar 12]. Disponível em: http://www.ibge.gov.br/estadosat/perfil.php?sigla=to Lista?

c Programa das Nações Unidas para o Desenvolvimento; Instituto de Pesquisa Econômica Aplicada; Fundação João Pinheiro. Atlas do desenvolvimento humano no Brasil 2013. Brasília (DF); 2013 [citado 2014 mar 12]. Disponível em: http://atlasbrasil.org.br/2013/

d Ministério da Saúde. Portaria nº 3.125, de 7 de outubro de 2010. Aprova as diretrizes para vigilância, atenção e controle da hanseníase. *Diario Oficial Uniao.* 27 mar 2010; Seção 1:55-60.

e Ministério da Saúde. Coordenação Geral de Hanseníase e Doenças em Eliminação. Banco de dados concedido por solicitação no portal da transparência. Brasília (DF); 2013 [citado 2014 mar 12]. Disponível em: http://tabnet.datasus.gov.br/cgi/deftohtm.exe?ibge/cnv/popto.def

f World Health Organization, Regional Office for South-East Asia. Enhanced global strategy for further reducing the disease burden due to leprosy (plan period: 2011-2015). New Delhi; 2009 [citado 2015 mai 9]. Disponível em: http://www.searo.who.int/entity/global_leprosy_programme/documents/enhanced_global_strategy_2011_2015.pdf

g Ribeiro FA. A invenção do Tocantins: memória, história e representação [dissertação]. Goiânia: Faculdade de Ciências Humanas e Filosofia da Universidade Federal de Goiás; 2001.

h Andrade RP. Conceitos de progresso e natureza na construção da Belém-Brasília. In: Anais do 13 Seminário Nacional de História da Ciência e Tecnologia; 2012; São Paulo, Brasil. Rio de Janeiro: Sociedade Brasileira da História da Ciência; 2012 [citado 2015 mai 9]. p.1-9. Disponível em: http://www.sbhc.org.br/resources/anais/10/1344996001_ARQUIVO_SBHC2012.pdf

i Ministério da Saúde, Secretaria de Vigilância em Saúde, Departamento de Vigilância das Doenças Transmissíveis. Plano integrado de ações estratégicas de eliminação da hanseníase, filariose, esquistossomose e oncocercose como problema de saúde pública, tracoma como causa de cegueira e controle das geohelmintíases: Plano de Ação 2011-2015. Brasília (DF); 2012 [citado 2015 mai 9]. (Série C. Projetos, Programas e Relatórios). Disponível em: http://bvsms.saude.gov.br/bvs/publicacoes/plano_integrado_acoes_estrategicas_2011_2015.pdf
